# Usability of a Tablet-Based Cognitive Assessment Administered by Medical Assistants in General Practice: Implementation Study

**DOI:** 10.2196/76010

**Published:** 2025-12-22

**Authors:** Philipp Schaper, Alexander Hanke, Stephan Jonas, Leon Nissen, Lara Marie Reimer, Florian Schweizer, Michael Wagner, Kristin Rolke, Carolin Rosendahl, Judith Tillmann, Klaus Weckbecker, Jochen René Thyrian

**Affiliations:** 1 Psychological Aging Research Department of Psychology University of Siegen Siegen Germany; 2 Department of Old Age Psychiatry and Cognitive Disorders University Hospital Bonn Bonn, North Rhine-Westphalia Germany; 3 Institute for Digital Medicine University Hospital Bonn Bonn, North Rhine-Westphalia Germany; 4 TUM School of Computation, Information and Technology Technical University of Munich Garching bei München, Bavaria Germany; 5 General Practice I and Interprofessional Care Institute of General Practice and Primary Care Witten/Herdecke University Witten Germany; 6 German Center for Neurodegenerative Diseases Greifswald Germany

**Keywords:** computerized assessment, dementia, digital assessment, digital platform, mild cognitive impairment, MoCA, Montreal Cognitive Assessment

## Abstract

**Background:**

Digital short cognitive tests administered by medical assistants (MAs) in general practitioners’ (GPs) practices have great potential for the timely identification of patients with dementia, because they can lead to targeted specialist referrals or to immediate reassurance of patients regarding their perceived concerns. However, integration of this testing approach into clinical practice requires good usability for the test itself, especially for cognitively impaired older adults.

**Objective:**

In this implementation study, the digital version of the Montreal Cognitive Assessment (MoCA) Duo was conducted by MAs in general practice. We tested if the interaction with the test is associated with usability problems for the patients and aimed to find additional relevant constructs that should be considered for the potential implementation of such digital tests into clinical practice. We focused the study on subjective success, usability, and workload as well as their association with the result of the cognitive test to assess whether the MoCA Duo can be implemented into general practice.

**Methods:**

In total, 10 GPs took part in the study. Within their practices, 299 GP patients (aged 51-97 years) with cognitive concerns completed the MoCA Duo administered by MAs. Subsequently, patients and MAs completed digital questionnaires regarding the interaction with the app. Usability was measured using the adapted System Usability Scale, and perceived workload using the National Aeronautics and Space Administration Task Load Index. For the perceived workload, we included an assessment of the patient by the MA. Results of the MoCA Duo were supplied to the GPs for their consultation with the patient.

**Results:**

The results indicated good usability for the MoCA Duo. Self-assessment by the patients indicated that 64% (191/299) could perform in the test to the best of their ability, affected by their MoCA score. We found significant higher usability ratings by patients with better MoCA scores as well as by younger patients. Furthermore, the perceived workload showed overall medium workload. We found significant correlations between the subjective perceived workload of the patients and the assessment by MAs. Self-assessments as well as assessments by the MAs were significantly influenced by the MoCA scores and the age of the participants.

**Conclusions:**

The results indicate good usability of the digital MoCA within the sample, supporting the idea that the resulting scores are adequate to assess cognitive status without dependence on technological affinity. Furthermore, the results highlight the relevance of heterogenous samples for comparable evaluation studies, based on the significant effect of cognitive status and age on usability and workload.

## Introduction

Based on the predictions by the Global Burden of Disease 2019 Dementia Forecasting Collaborators, more than 57.4 million people globally were living with dementia in 2019, with an expected increase to 152.8 million in 2050 [1,2]. Even though most of the predicted future cases will be in developing countries, an increase in cases is also expected for industrialized countries. In the case of Germany, in addition to the existing 1.8 million cases, an additional 360,000 to 440,000 new cases are expected [2]. The high number of cases in itself is a challenge for public health. However, due to the progressive nature of dementia, timely diagnosis severely affects intervention and potential treatment options, which currently only slow progression of the disease. Therefore, timely diagnosis mediates the personal, medical, social, and economic impact of the disease [3]. For the best results, it is crucial that testing for early signs is accessible within standard health care [4].

The German guideline on dementia already recommends the administration of cognitive short tests such as the Montreal Cognitive Assessment (MoCA) or the Mini Mental State Examination (MMSE) if self-reported memory problems are indicated by the patient or in response to the general practitioner’s (GP) request [5]. This is due to the gatekeeping role of GPs in German health care, where they not only treat patients but also function as coordinators for further treatment by specialists, including neurologists in the case of dementia symptoms. However, within general practice, patients with manifested dementia are often not recognized as such [6,7] due to no or insufficient testing [8], resulting in delayed diagnosis and belated management of the disease. The rate of affected patients without an adequate diagnosis could be as high as 50% [9]. Due to the late diagnosis, patients are often referred to specialists once the symptoms are severe and advanced diagnostics are no longer indicated. In contrast, patients benefit most if they receive a timely diagnosis [10].

There are multiple reasons for the lack of timely diagnosis, including the high workload in GP practices, lack of expertise with mild cognitive impairment (MCI) tests, and low monetary compensation for the current diagnostic methods [11,12]. These problems have already resulted in many GPs withdrawing from dementia diagnostics altogether [13]. Even though such structural problems cannot be changed rapidly, novel assessment approaches involving supervised or unsupervised digital cognitive testing could benefit patients as well as GPs [14,15].

The inclusion of digital cognitive testing into standard processes within GP practices allows for a more approachable and standardized testing [16,17]. In turn, GPs have an additional indicator or a starting point for dementia diagnostics, which helps to recognize initially mild symptoms [10]. This would lead to more targeted referrals to specialists because patients are identified early enough to benefit from their timely diagnosis in terms of treatment or at least disease management, while healthy patients and severe cases of dementia receive no superfluous extended diagnostics. It has been shown that using IT-supported assessments is perceived as beneficial by professional users [18] and in the context of GP practice [19].

However, the relatively sudden increase in apps within the German health care system [20], including several options for dementia screening [21,22] or care, results in apps not being used at all, different preferences between practitioners and patients, and missing standardization of the process [23]. Even though their potential benefit is recognized in theory, the integration of apps within primary care is still lacking [24]. Therefore, it is necessary not only to evaluate the validity of a cognitive screening app within an experimental study but also whether the app and the resulting procedure are suited for integration into general practice.

This study focuses on the quantitative usability evaluation of the iCreate study (“Digitally supported case finding to improve the diagnosis and care of patients with dementia in primary care”), a study that aims to assess the feasibility, validity, usability, and clinical impact of tablet-based cognitive testing by medical assistants (MAs) in GP practices. This implementation study compares the patient’s interaction with the digital cognitive test against established usability criteria, as well as the perceived workload for the patients with that reported by the MAs. The results should allow assessment of whether the test can be integrated into general practice and highlight potential problems as well as avenues for further research.

## Methods

### Overview

This study addresses the usability aspects of the tablet-based MoCA Duo within the iCreate study, a prospective feasibility study of tablet-based MoCA testing administered by MAs in GP practices. Producing meaningful data in digital dementia diagnostics requires that the testing process as well as the results are not influenced by usability problems [25].

### Study Procedure

The study took place from June 1, 2023, to March 3, 2025, and was conducted by 4 university institutes and 1 research institution in North Rhine-Westphalia, Germany. Potential GPs in the city of Bonn and the Rhine-Sieg district in western Germany were contacted by the study team and offered participation in the project until the criterion of integrating 10 GPs, as set by the overarching iCreate study, was met.

Each GP practice was visited at least twice for presentation of the study and for a 1-hour training of the MAs to administer the tablet-based MoCA Duo. Patients were informed by posters and flyers in the GPs’ waiting room about optional cognitive testing in case of concerns regarding cognition and either requested this test themselves or followed the suggestion of the GPs or MAs to examine a suspected impairment. Integration into the study was therefore not randomized but rather based on selection by the participants themselves or the GP practice team.

The MoCA Duo is a 1:1 app replication of the paper-and-pencil MoCA [26], which is used in clinical practice, and good equivalence of both MoCA versions has been established [27]. The app and all subsequent questionnaires were completed on an iPad Air (11-inch, Apple Inc). Study participation including consent forms, the MoCA Duo, and all questionnaires, took approximately 25 minutes. The GP practices were compensated for the additional time and effort to conduct the test (15€ [US $17.36] for the MAs, 25€ [US $28.94] for the practice).

The MoCA Duo creates a result summary, which was supplied to the GP, who suggested an adequate next step, ranging from no further procedures to a retest in the GPs office in a year, or additional testing and diagnostic workup in the memory clinic of the University Clinic Bonn. Results of qualitative interviews with a smaller set of GPs, Mas, and patients have recently been published [28]. The interviews indicated positive reception of the digital test from all 3 groups, and GPs and MAs indicated interest in long-term implementation into standard practice. Results on diagnostic accuracy (prediction of MCI or dementia diagnoses in subsequent memory clinic assessment) will be reported elsewhere.

### Questionnaires

First the MoCA Duo was administered by the MAs to assess the cognitive status of the patient. Subsequently, the patients used the same tablet to answer questionnaires on usability, workload, and secondary measures regarding the MoCA Duo. Finally, the MA completed an assessment on the perceived workload of the patient. There were no additional questionnaires for the MA. Participants were free not to take part in the study; however, there were no aborts during testing, and therefore only complete data for all measures could be used.

#### Cognitive Status

To assess cognitive impairment, we used a commercially available digital adaptation of the MoCA [29,30], which directly imitates the paper version of the test [27], with instructions for the administering MA as well as for the patient. Two cutoffs were used based on large German-speaking normative and clinical samples [31]. A score of 23 or lower indicates high risk for MCI, a score of 27 or more indicates low risk for MCI, and scores in between require further examination, subsequently referred to as “within threshold.”

#### Usability

The System Usability Scale (SUS) is an established measure used to assess the user’s subjective perception of an interface [32] and has been previously validated [33]. For this study, we used the already published simplified version of the questionnaire, especially designed for cognitively impaired and older adults based on the expected sample population [34]. This scale uses similar but slightly reworded statements, which refer to “confusion” caused by the system rather than to “inconsistency.” As in the original version, the items are presented as statements to be rated on a Likert scale from 1 to 5, ranging from “strongly disagree” to “strongly agree.” The German translation of the original SUS by Gao et al [35] was used but had to be slightly adapted by the authors to reflect the adaptations by Holden [34]. The resulting score, between 0 and 100, is subsequently categorized into different categories (Bangor et al [32], as applied by Souza et al [36]): below 20.5 (worst imaginable), 21 to 38.5 (poor), 39 to 52.5 (average), 53 to 73.5 (good), 74 to 85.5 (excellent), and 86 to 100 (best imaginable).

#### Workload

The National Aeronautics and Space Administration Task Load Index (NASA TLX) is an established measure to assess the workload of a task or interaction in regard to mental, physical, and temporal demand as well as performance, effort, and frustration [37]. The scale can be used regarding one specific task, which necessarily interrupts the interaction, or for an entire process. The instrument has been used in diverse contexts, often for work-related tasks, but also for workloads in older patients in interaction with health care instruments [38,39]. In addition to the commonly used self-assessment for the scale, we also asked the MAs to rate the respective patients on the same scales regarding their interaction with the MoCA Duo. In contrast to the SUS, the resulting scores of the NASA TLX do not result in predefined categories; however, differences in the subscales can pinpoint the source of the workload. Furthermore, the scale is sensitive to small differences in workload when comparing different applications [39], therefore creating a meaningful baseline for subsequent research.

#### Secondary Variables

We added additional questions for more demographic information, such as age, relationship status, and whether living on one’s own, but also addressed the self-assessed performance of the patients within the MoCA Duo, as well as their daily use of technology. For assessment of their own performance, participants were asked if they could show their full ability within the test. If not, participants could choose from a range of potential reasons or could indicate one in free text.

To assess daily use of technology, participants were asked how often they used a smartphone or tablet in a day, with the options of “never,” “rarely,” “regularly,” and “often.” We also assessed if they had prior experience with cognitive tests. As an additional indicator for the usability of the digital MoCA, we asked the participants to compare the interaction with the test relative to their interaction with their own smartphone or tablet, with the options of “easier,” “similar,” “more difficult,” and “I don’t use a similar device.”

### Data Analysis

To test the plausibility of our results, we plan an ANOVA for the scores in the MoCA using the age groups as a factor, expecting a decline in the scores indicating increasing risk with higher age. Subsequently, we plan an analysis of the usability assessment based on the categories of the MoCA to test if usability is affected by cognitive status. For the workload, we compare self-assessment and assessment by the MAs using correlations. The different subscales of the self-assessment will be analyzed using multivariate analysis of variance (MANOVA), using the age groups in one analysis and the categories of the MoCA in a separate analysis as factors. These analyses will also be repeated for the assessment by the MAs.

Because low affinity with technology might be associated with worse results in a digital MoCA [40], a chi-square test on the daily smartphone use of the patients and the 3 categories for the MoCA scores is conducted.

All analyses were conducted using R software (version 4.0.0; R Foundation for Statistical Computing).

### Ethical Considerations

There were no invasive procedures involved in the study. The instruments were developed and administered as efficiently as possible to keep time demands low. The Ethics Committee of the Medical Faculty of the University of Bonn, Germany (No 258/23-EP), granted approval for the whole iCreate feasibility study. All participants gave written informed consent based on a study description and explanation of data use in German. Study data were clearly indicated as deidentified. The GP practices were compensated for the additional time and effort to conduct the test (€15 [US $17.36] for the MAs, €25 [US $28.94] for the practice); the participants themselves received no additional compensation for study participation.

## Results

### Sample

#### Medical Practices

Overall, a total of 10 GPs in the city of Bonn and the Rhine-Sieg district in western Germany took part in the study. These practices tested between 4 and 70 patients during the study period.

#### Patients

The sample consisted of 299 patients who decided to partake and agreed to the use of the MoCA Duo. The participants were dominantly female (193/299, 65%), had a mean age of 73.23 years (SD 9.82; range 51-97 years), mostly lived with someone else (206/299, 69%), and were mainly in a relationship (204/299, 68%). Based on participants’ recollection, a total of 13% (40/299) had prior experience with cognitive tests, 6% (17/299) were uncertain, 80% (238/299) reported not having prior experience, and 1% (4/299) chose not to answer the question.

### Cognitive Status

The mean score in the MoCA for the sample was 23.21 (SD 5.22). According to the thresholds by Thomann et al [31], our sample contained 33% (100/299) patients with low risk for MCI, 43% (130/299) patients with high risk for MCI, and 23% (69/299) within the threshold. The MoCA scores based on equally spaced age groups are presented in Table 1. A 1-way ANOVA showed a significant decline in MoCA scores with increase in age (*F*_2_=28.84; *P*<.001).

**Table 1:** Mean values and SDs for usability and workload based on age groups.

Scale and subscale		Age groups (years), mean (SD)			
		50 to 60 (n=29)	61 to 70 (n=87)	71 to 80 (n=96)	81 and older (n=87)
MoCA^a^ scores		26.21 (3.37)	25.97 (3.43)	22.73 (4.91)	19.99 (5.58)
SUS^b^		76.03 (18.52)	77.56 (16.97)	66.20 (20.77)	57.76 (20.75)
**NASA TLX>^c^ self-assessment**					
	Mental workload	6.55 (5.42)	6.70 (5.15)	8.10 (5.40)	8.70 (5.81)
	Physical workload	3.21 (3.88)	2.89 (3.64)	4.26 (4.72)	5.14 (5.74)
	Temporal workload	4.45 (4.28)	4.09 (4.75)	4.54 (4.36)	5.75 (5.41)
	Performance	13.07 (5.69)	13.29 (5.31)	11.09 (5.34)	8.94 (4.62)
	Effort	8.72 (5.01)	7.78 (4.91)	8.55 (4.91)	8.68 (5.62)
	Frustration	6.62 (5.31)	6.24 (5.62)	7.35 (5.91)	7.36 (6.01)
**NASA TLX external assessment**					
	Mental workload	5.76 (5.69)	5.91 (5.29)	8.64 (5.98)	11.78 (6.05)
	Physical workload	2.79 (3.82)	3.43 (4.80)	4.71 (4.73)	5.83 (6.12)
	Temporal workload	4.66 (5.07)	5.17 (5.04)	5.59 (4.77)	6.17 (5.54)
	Performance	13.24 (6.15)	13.80 (6.05)	11.33 (5.82)	8.00 (5.50)
	Effort	6.10 (5.17)	7.21 (5.80)	9.33 (5.68)	11.71 (5.92)
	Frustration	5.21 (5.21)	6.16 (5.81)	8.45 (6.26)	8.49 (6.30)

^a^MoCA: Montreal Cognitive Assessment.

^b^SUS: System Usability Scale.

^c^NASA TLX: National Aeronautics and Space Administration Task Load Index.

### Usability

The mean usability rating on the SUS was relatively high (mean 68.00, SD 21.02) within the possible range from 0 to 100. Based on the benchmarks [32] added to the original SUS scale [41], the digital test would be considered to show good usability, falling in the range of 53 to 73.5. The overall distribution of results is shown in Multimedia Appendix 1.

Table 2 presents the SUS scores relative to the categories for the MoCA. A 1-way ANOVA resulted in a significant effect of the MoCA categories on the SUS score (*F*_2_=62.79; *P*<.001), with lower scores in the threshold category and even lower scores in the high-risk category. The SUS scores relative to equally spaced age groups in Table 1 show a comparable pattern, with a 1-way ANOVA indicating a significant main effect (*F*_3_=16.85; *P*<.001), with lower scores for older patients.

**Table 2:** Mean values and SDs for usability and workload relative to the 3 Montreal Cognitive Assessment (MoCA) categories.

Scale and subscale		Low risk for MCI^a^ (n=100), mean (SD)	Within threshold (n=69), mean (SD)	High risk for MCI (n=130), mean (SD)
SUS^b^		81.18 (16.52)	72.54 (16.74)	55.46 (18.95)
**NASA TLX>^c^ self-assessment**				
	Mental workload	6.14 (4.93)	5.83 (4.28)	9.94 (5.72)
	Physical workload	2.54 (3.24)	2.93 (3.84)	5.72 (5.59)
	Temporal workload	3.56 (3.93)	3.86 (4.21)	6.14 (5.38)
	Performance	14.82 (5.03)	11.39 (4.97)	8.54 (4.29)
	Effort	6.78 (4.75)	6.87 (4.15)	10.42 (5.19)
	Frustration	4.88 (5.01)	6.41 (5.44)	8.85 (5.96)
**NASA TLX external assessment**				
	Mental workload	4.20 (4.85)	7.97 (5.17)	12.04 (5.54)
	Physical workload	2.50 (3.76)	3.42 (4.43)	6.55 (5.78)
	Temporal workload	3.43 (3.86)	5.54 (4.51)	7.18 (5.64)
	Performance	16.65 (4.72)	12.42 (4.54)	6.52 (4.04)
	Effort	5.06 (4.92)	8.58 (4.74)	12.47 (5.45)
	Frustration	3.84 (4.66)	7.07 (5.27)	10.50 (6.05)

^a^MCI: mild cognitive impairment.

^b^SUS: System Usability Scale.

^c^NASA TLX: National Aeronautics and Space Administration Task Load Index.

### Workload

Overall mean ratings for the self-assessment of the patients and the assessment of the patients by the MAs are shown in Table 3. The self-assessment on all 6 subscales showed significant positive correlations with the external assessment in a range from 0.44 to 0.57. The lowest scores occurred for physical and temporal demand, and the highest for self-assessed performance.

Tables 1 and 2 present self-assessment and external assessment relative to the categories of the MoCA (Table 2) and relative to the equally spaced age groups (Table 1). A MANOVA for all self-assessments showed an overall significant main effect of the MoCA categories on the subscales (*F*_12,584_=9.93; *P*<.001), with all scores rising from patients with low risk for MCI to patients with high risk for MCI, with the exception of the performance subscale, which showed an opposing trend. A MANOVA for all external assessments also showed a significant effect (*F*_12,584_=20.18; *P*<.001) and the same pattern in the descriptive values.

A MANOVA on all self-assessments regarding the age groups showed an overall significant main effect of the age groups on the subscales (*F*_18,876_=2.53; *P*<.001), with all scores rising from younger to older patients, except for the performance subscale, which showed an opposing trend. A MANOVA for all external assessments also showed a significant effect (*F*_18,876_=4.38; *P*<.001) and the same pattern in the descriptive values.

**Table 3:** Mean values and SDs of self-assessment and external-assessment of National Aeronautics and Space Administration Task Load Index (NASA TLX) subscales including correlations of the assessments.

NASA TLX^a^ subscale^b^	Self-assessment, mean (SD)	External assessment, mean (SD)	Correlation, *r*	*P* value
Mental workload	7.72 (5.50)	8.48 (6.24)	0.44	<.001
Physical workload	4.01 (4.76)	4.47 (5.21)	0.55	<.001
Temporal workload	4.75 (4.82)	5.55 (5.11)	0.54	<.001
Performance	11.3 (5.43)	11.3 (6.25)	0.57	<.001
Effort	8.38 (5.13)	9.09 (6.04)	0.44	<.001
Frustration	6.96 (5.79)	7.48 (6.15)	0.57	<.001

^a^NASA TLX: National Aeronautics and Space Administration Task Load Index.

^b^Values for the scores range from 1 to 20.

### Secondary Variables

We found that 64% (191/299) of participants felt unrestricted in displaying their ability within the test. Of those who could not perform to the best of their ability, 41% (44/108) reported agitation as the main reason, 27% (29/108) thought the unusual tasks hindered them, and 17% (18/108) felt uncomfortable with being in a test situation. Additionally, 34% (37/108) reported other reasons in free text, which included anxiety, the language barrier, and physical ailments. The remaining categories were rarely selected and included 8% (9/108) problems understanding specific tasks and 1% (1/108) problems with the interaction.

Within the categories of the MoCA, patients within the group with low risk for MCI could show their full potential in 84% (84/100) of cases, for the within-threshold group this value dropped to 59% (41/69) and was lowest in the high-risk-for-MCI group with 51% (66/130).

**Figure 1:**
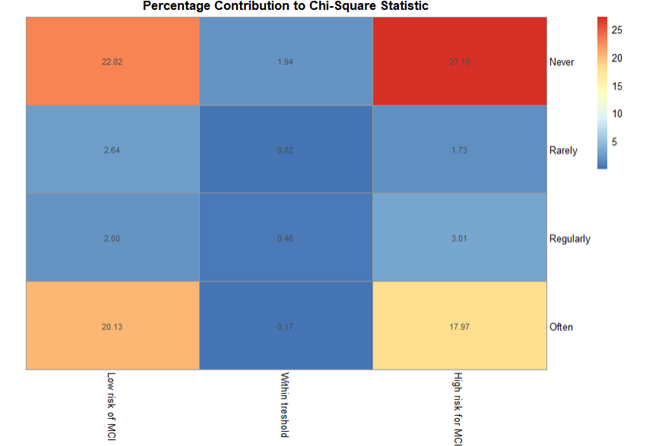
Percentage contribution of affinity with technology and Montreal Cognitive Assessment (MoCA) categories. Affinity to technology is indicated by smartphone or tablet use on a daily bases, with “never” indicating low affinity to technology and “often” indicating high affinity to technology. MCI: mild cognitive impairment.

Concerning affinity to technology, the sample was heterogenous, with 24% (72/299) reporting using their smartphone or tablet often in one day, 32% (97/299) using it regularly, 21% (63/299) using it rarely, and 22% (67/299) never using it. Table 4 presents the number of patients within the categories regarding cognitive status relative to their technology affinity. A subsequent chi-square test on technology affinity and the 3 categories for the MoCA scores indicated a significant association between both measures (N=299; *χ*²_6_=66.3; *P*<.001). Figure 1 shows the percentage contribution of deviation to the statistic, highlighting two main patterns (1) for patients with low technology affinity, the count in the low-risk group is very low, while it is high in the high-risk group (top row, columns 1 and 3); and (2) for patients with high technology affinity, the count is high in the low-risk group while it is low in the high-risk group (bottom row, columns 1 and 3).

**Table 4:** Cross table on technology affinity and categories on the Montreal Cognitive Assessment (MoCA) score.

Technology affinity	Low risk for MCI^a^, n (%)	Within threshold, n (%)	High risk for MCI, n (%)
Never	4 (9)	11 (16)	52 (40)
Rarely	15 (15)	15 (22)	33 (25)
Regularly	39 (39)	25 (36)	33 (25)
Often	42 (42)	18 (26)	12 (9)

^a^MCI: mild cognitive impairment.

Regarding the comparison of the interaction with the app relative to their own device, a total of 31% (94/299) of patients considered the digital MoCA simpler, 33% (99/299) considered it of similar difficulty, 8% (23/299) found it more difficult, and 28% (83/299) reported not having a smartphone or tablet for comparison.

## Discussion

### Overview

In this evaluation study on the usability of the integration of a digital MoCA test to be conducted by MAs within GP practices, we found overall positive results. Our analyses show the good usability of the digitalized MoCA and support its feasible integration into the routine care setting in GP practices. As outlined in the review on mobile technology for cognitive assessment by Koo and Vizer [42], usability studies have to include patients who are cognitively impaired in addition to healthy controls. This study presents (to our knowledge) the first usability evaluation on a heterogenous sample including patients who are cognitively impaired for the MoCA Duo [27].

### Findings

Within a heterogenous sample in terms of MoCA scores, that is, potential cognitive impairment, as well as different affinity to technology, we found that most patients could show their full potential (191/299, 64%) within the cognitive test while indicating good usability based on the SUS scores. Our analyses indicated significantly lower scores regarding usability for patients with higher risk for MCI, as well as for older patients. Both workload assessments were also significantly influenced by both factors. This further highlights the relevance of including patients who are cognitively impaired and older in evaluation studies.

The instances in which patients reported not being able to perform to the best of their ability were (at least in the self-assessment) not based on the usability or the fact that the test was digital, but rather on factors such as anxiety, which would also be relevant in the paper-based version. However, a study addressing this potential link found no indication of a detrimental effect of anxiety [43]. However, it is also noteworthy that within the group of patients with high risk for MCI the self-report of being able to perform to the best of their ability was lowest (66/130, 51%) compared to both other groups.

The assessment of the workload within the test showed a plausible pattern of results. We found high but not excessive mental workload within the scales for mental demand and effort and low physical demand. The self-assessment of performance was descriptively high, which appears understandable given how easy it is within the tasks of the MoCA to assess one’s own performance. Because there are no time restrictions in the MoCA tasks, the resulting perceived time pressure was low. Critically, the assessment of the workload by the MA was positively correlated with the self-assessment of the patients. Besides the additional confidence in the results of the patients, this supports the role of MAs within GP practices, because their assessment, even though it might be informal, could serve as an additional indicator for subsequent treatment by the practitioner.

Considering the decline in SUS scores, the lower self-report of ability to perform to the best of one’s own ability, and the increase in workload with increasing risk for MCI and age, it must be considered if the usability problems indicate problems with the app or rather the result of the cognitive decline. Given the present results, we suggest that the usability for the MoCA Duo is high for people not significantly cognitively challenged by the test, but with insufficient cognitive resources for the tasks, the usability is perceived as lower, indicated by the increased workload.

The diagnostic value of the paper-based MoCA has been demonstrated across different languages [44], and comparisons between digital and paper-based versions show high consistency [27], except in instances where physical interaction is necessary [40]. This highlights the relevance of taking experience with technology into account but also demonstrates the relevance of good usability in digital applications, especially for patients without experience with the respective technology.

We found a significant relationship between the MoCA assessment and the daily use of a smartphone or tablet as an indicator of technological affinity, the main differences in observed and expected values were especially prominent in the edges of the 3 × 4 table in Figure 1, with higher-than-expected values for patients with low risk for MCI often using their smartphone, as well as for patients with high risk for MCI never using such a device. Conversely, fewer-than-expected observations were made for patients with low risk for MCI never using a smartphone and patients with high risk for MCI using it often. Even though unfamiliarity with the technology might have a negative impact on the MoCA score [40], this would only account for the increase of patients who never use a smartphone with low MoCA scores. Furthermore, technology use has been previously shown as a predictor for MoCA scores [45]. Therefore, we suggest that the pattern of results might be better explained by patients with healthy cognition being more able and interested in learning and subsequently using smartphones, fitting the significant decline in MoCA scores with age. However, further study regarding this link is still necessary.

### Limitations

Some limitations must be considered for this study. Even though the high number of participants is encouraging, there is likely to be a selection bias regarding which patients took part in the study due to subjective cognitive problems or based on the offer in GP practices. Additionally, self-selection to not participate in the study was not recorded and might have influenced the final sample and the subsequent results. This aspect must be addressed in future work in terms of an evaluation study assessing the applicability of the approach in standard health care.

Based on the large and diverse sample in this study, we do not expect issues regarding usability in future work. Other potential barriers such as proficiency in German, deficits with eyesight, or motor problems (ie, tremors) would have to be considered when administering the test. Due to the setting of the study, the validity of the MoCA scores was not tested against other measures, but based on the validation study by Klil-Drori et al [30] and the plausible patterns of our secondary measures, we do not expect severe deviations from a paper-based MoCA.

### Future Work

Future work should address the reported effects of technological affinity and the MoCA scores, especially regarding the digital assessment of the test. Finally, an evaluation of the integration of the cognitive test by MAs and the subsequent integration of the results into GP practices into standard health care should be conducted. Ideally, a standardized training module would be used to standardize the application of the test by MAs.

Overall, this implementation study further supports the positive outlook of integrating digital cognitive testing by MAs into GP practices as outlined by the results of other published work of the iCreate project, such as the results of qualitative interviews with practitioners, MAs, and patients. The ease of data collection across several GP practices also highlights the benefit of digital testing; this is further highlighted by the automatic analysis of the results, which allowed discussion of the performance immediately following the test. If adopted in standard health care, the test result should also be available to the specialist in case of further diagnostic testing, removing the need to repeat tests.

## Appendix

Multimedia Appendix 1 Distribution of System Usability Scale (SUS) scores.

## Abbreviations

GP: general practitioner
MA: medical assistant
MANOVA: multivariate analysis of variance
MCI: mild cognitive impairment
MMSE: Mini-Mental-State Examination
MoCA: Montreal Cognitive Assessment
NASA TLX: National Aeronautics and Space Administration Task Load Index
SUS: System Usability Scale

## Data Availability

The datasets generated or analyzed during this study are not publicly available, as this has not been specifically allowed in the written informed consent of the participants, but they are available from the corresponding author on reasonable request.
