# Supplementary figures and images for: Usability of a Tablet-Based Cognitive Assessment Administered by Medical Assistants in General Practice: Implementation Study
Source: JMIR Form Res. 2025 Dec 22;9:e76010. doi: 10.2196/76010 (PMC12770926; doi:10.2196/76010)

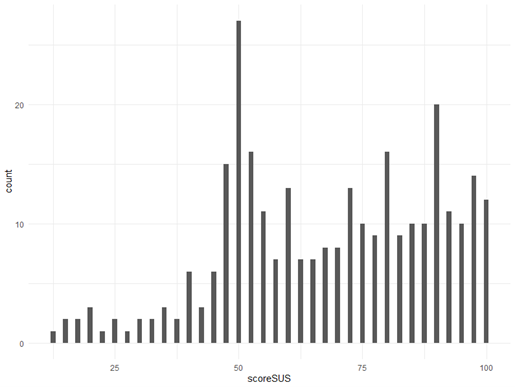

Supplement: Multimedia Appendix 1 [file formative_v9i1e76010_app1.png]
